# Protein kinase C and cardiac dysfunction: a review

**DOI:** 10.1007/s10741-017-9634-3

**Published:** 2017-07-12

**Authors:** Raphael M. Singh, Emanuel Cummings, Constantinos Pantos, Jaipaul Singh

**Affiliations:** 1 School of Forensic and Applied Sciences, University of Central Lancashire, Preston, England PR1 2HE UK; 2 Faculty of Medicine and Health Sciences, University of Guyana, Turkeyen, Georgetown, Guyana; 3 Department of Pharmacology, School of Medicine, University of Athens, Athens, Greece

**Keywords:** Protein kinase C, Heart failure, Hypertrophy, Fibrosis, Cardiac remodelling

## Abstract

Heart failure (HF) is a physiological state in which cardiac output is insufficient to meet the needs of the body. It is a clinical syndrome characterized by impaired ability of the left ventricle to either fill or eject blood efficiently. HF is a disease of multiple aetiologies leading to progressive cardiac dysfunction and it is the leading cause of deaths in both developed and developing countries. HF is responsible for about 73,000 deaths in the UK each year. In the USA, HF affects 5.8 million people and 550,000 new cases are diagnosed annually. Cardiac remodelling (CD), which plays an important role in pathogenesis of HF, is viewed as stress response to an index event such as myocardial ischaemia or imposition of mechanical load leading to a series of structural and functional changes in the viable myocardium. Protein kinase C (PKC) isozymes are a family of serine/threonine kinases. PKC is a central enzyme in the regulation of growth, hypertrophy, and mediators of signal transduction pathways. In response to circulating hormones, activation of PKC triggers a multitude of intracellular events influencing multiple physiological processes in the heart, including heart rate, contraction, and relaxation. Recent research implicates PKC activation in the pathophysiology of a number of cardiovascular disease states. Few reports are available that examine PKC in normal and diseased human hearts. This review describes the structure, functions, and distribution of PKCs in the healthy and diseased heart with emphasis on the human heart and, also importantly, their regulation in heart failure.

## Heart failure

Cardiovascular diseases (CVDs) are composed of several different pathologies, including coronary ischemic heart disease, rheumatic heart disease, congenital cardiovascular defects, high blood pressure, heart failure, stroke, arrhythmias, myocardial infarction, and diseases of the arteries including endothelial dysfunction and atherosclerosis. Despite significant progress in the prevention and treatment of CVDs, statistics indicate that CVDs are the leading cause of deaths throughout the world [1]. The World Health Organization (WHO) estimates that CVDs are responsible for 17.5 million deaths in 2012, representing 31% of all global deaths. Of these, 7.4 million died of ischaemic heart disease and 6.7 million from stroke.

According to the American Heart Association [2], CVDs accounted for 31.3% (786,641) of all deaths (total of 2,515,458) in 2011. On the basis of 2011 death rate data, mortality owing to CVDs accounted an astounding 2150 people dying daily with an average of 1 death every 40 s.

Heart failure (HF) is a clinical syndrome characterized by impaired ability of the left ventricle to either fill or eject blood [3]. American Heart Association (AHA) statistical update in 2015 reported that 1 in 9 deaths has HF mentioned on the death certificate and data from 2011 revealed that HF any-mention mortality was 284,388 (129,635 males and 154,753 females). In 2012, total cost for HF was estimated to be $30.7 billion, of which a total of 68% was attributed to direct medical cost. Projection shows that by 2030, the total cost of HF will increase almost to $69.7 billion from 2012 in the USA [4].

HF can no longer be considered a simple contractile disorder or a disease of the heart alone. It is now accepted that as heart disease progresses into HF, heart size increases, cardiac function deteriorates, and symptoms of HF become evident. The aetiology of HF is diverse and it includes hypertension, myocardial infarction, arrhythmias, bacterial endocarditis, ischaemia, idiopathic and diabetic cardiomyopathy, coronary heart disease, and congenital cardiovascular defects. Of these aetiologies, coronary artery disease and myocardial infarction are the most common [5].

## Protein kinase C

### Discovery and structure

Protein kinases C (PKC) were identified over three decades ago, as kinases that are activated by proteolysis [6]. Initially identified as a nucleotide-independent, Ca^2+^-dependent serine kinase, PKCs are a family of serine/threonine kinases that are activated as a result of receptor-dependent activation of phospholipase C and the hydrolysis of membrane phosphoinositides [7]. PKCs are now known to be major mediators of signal transduction pathways and have been shown to regulate sets of biological functions as diverse as cell growth, differentiation, apoptosis, transformation, tumourigenicity, and others [8, 9].

According to differences in the binding capability of their regulatory domain, the presently known 13 members of the PKC family have been grouped into 3 classes: the classical PKCs (α, β1, β2, γ), novel PKCs (δ, ε, η, θ), and atypical PKCs (ζ, λ/ ι) [9, 10].

The first PKCs to be identified and cloned were α, β, and γ isozymes, initially isolated from brain complementary DNA (cDNA) libraries [11]. Low-stringency screening of brain cDNA libraries with probes derived from the α, β, and γ isozymes yielded three additional PKCs, the PKC-δ, PKC-ε, and PKC-ζ isozymes [12], and further low-stringency screens of other tissue cDNA libraries led to identification of PKC-η [13], PKC-θ [14], and PKC-ι (the mouse ortholog of λ in humans) [15]. At present, there are over 450 protein kinases in the human genome [16].

**Fig. 1:**
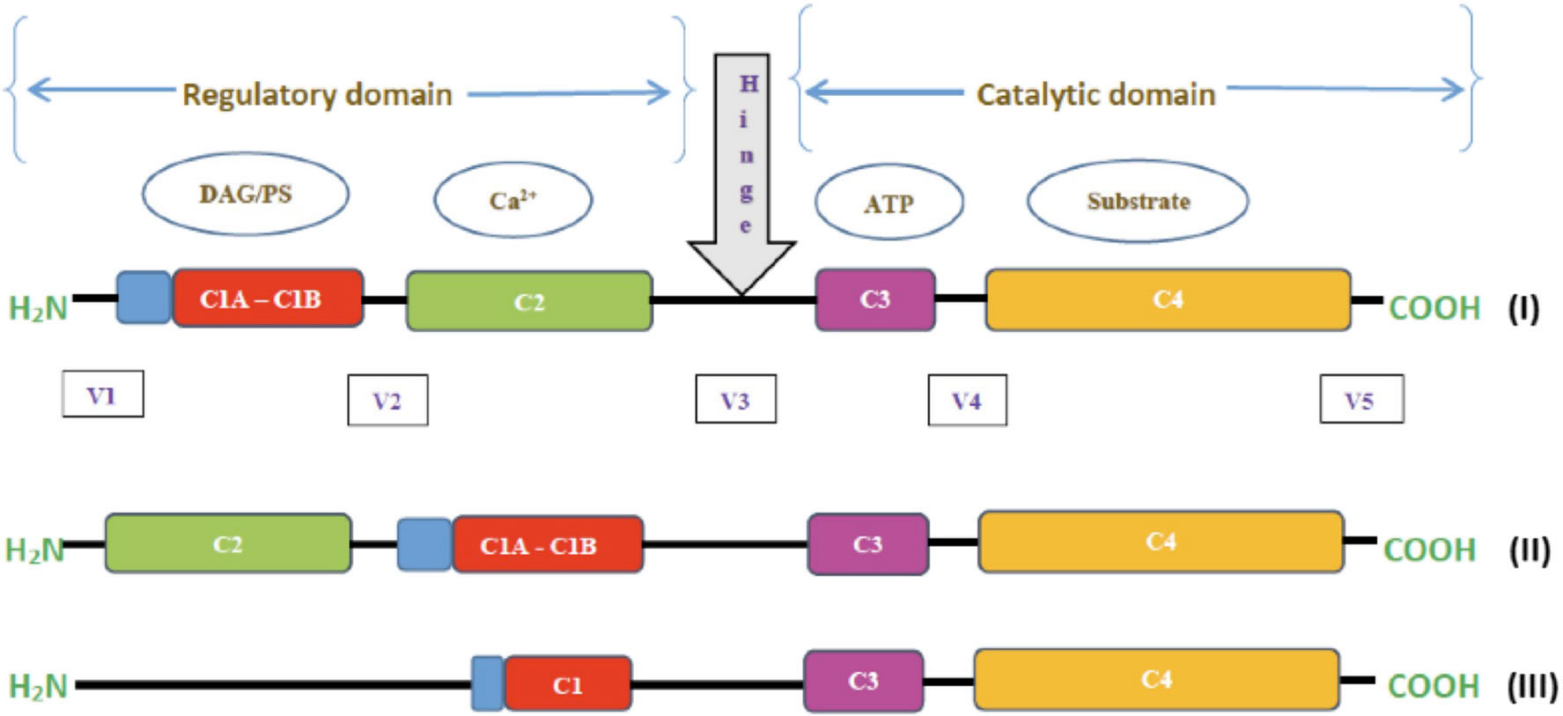
Schematic representation of the primary structure of PKC gene family. PKC isoenzymes are composed of single polypeptide chains that consist of regulatory and catalytic domains. Indicated are a series of conserved (*C1–C4*) regions and variable regions (*V1–V5*). The C1 region (*red*) consist of a cys-rich motifs, C2 (*green*) is the calcium binding region, C3 (*purple*) comprises the ATP binding lobe, and C4 (*gold*) is the substrate binding lobe. Also indicated is the pseudosubstrate domain (*blue*) in the V1 region. The regulatory and catalytic domains are separated by V3 (*hinge*). Structure *(I)* represents cPKC: α, β1, β11, γ, structure *(II)* represents nPKC: δ, ε, η, θ, and structure *(III)* represents aPKC: ζ, λ/

All PKCs have a common general structure composed of a single polypeptide chain with two principal modules including a NH_2_-terminal regulatory domain that contains the membrane-targeting motifs and a COOH-terminal catalytic domain that binds ATP and substrates (see Fig. 1). Initial research in 1986 by Caussens et al. [11] revealed that throughout the primary sequence of the enzymes, there are four conserved (C1–C4) regions, with each region being a functioning module, and are flanked by variable (V) regions.

### cPKCs (α, β1, β2, γ)

The classical PKC consists of five variable and four conserved regions (C-regions). The catalytic central part is found in the C4 region; the C3 region contains the ATP binding site. The C2 region contains the recognition site for acidic lipids and also, it is responsible for binding (Ca^2+^), while the C1 region is responsible for diacylglycerol or phorbolester (e.g. phorbol-12,13-myristate-acetate (PMA)) binding and consists primarily of two cysteine-rich ‘zinc-finger-like’ regions. The activity of this group depends on Ca^2+^ and on the presence of phospholipids (DAG) and phosphatidylserine.

### nPKCs (δ, ε, η, θ)

For the novel PKCs, they are structurally similar to the conventional cPKCs. However, the C2 region does not have functional groups to mediate Ca^2+^ binding and thus, it does not depend on Ca^2+^, but requires dioleoylglycerol and phatidylserine for their activation.

### aPKCs (ζ, λ/ι)

The atypical PKCs are the third group of isozymes and these differ significantly in structure from the previous two groups. The C1 region contains only one of the cysteine-rich motif and the C2 region is absent. These isozymes, therefore, do not depend on Ca^2+^ for activation and they also lack sensitivity to dioleolglycerol/phorbolesters. Research has further shown that these isozymes are targets of lipid-derived secondary messengers [17] and may be activated by lipids such as arachidonic acid and phosphatidylinositol 3,4,5-triphosphate. Initial studies by Nishizuka [9] revealed that protein kinase C was involved in lipid signalling for sustained cellular responses. The catalytic and regulatory halves in PKCs are separated by a hinge region that is proteolytic [18] which results in a constitutively active kinase [6]. Further detailed works on PKC structure are described in other studies [19–22].

### Regulations

PKCs are central enzymes in the regulation of cell growth and hypertrophy and play a major role in signal transduction in the heart. Initial work, mostly using phorbol esters, showed that PKC is a critical enzyme in regulation of cell growth and differentiation [23], in the phosphorylation of substrates [24], in stimulation of other proteins such as kinases [25], in the regulation of ion channel and receptors [26], and altered gene expression [27]. It has been reported that PKC activation plays a critical role in the development of delayed preconditioning by translocating to the perinuclear region to induce gene expression or by activating mitogen-activated protein kinases (MAPK). Although these initial studies were significant, phorbol esters are not izozyme-selective and therefore, it was not possible to identify which isozymes regulate a given function.

Intracellular events, associated with response to circulating hormones, trigger activation of PKC. These events can influence various physiological processes in cardiovascular system, resulting in chronotropic and inotropic effects [28]. Numerous studies based on animal models have implicated PKC activation with a number of cardiac diseases and heart failure, with much of the initial focus being placed on cardiac ischaemia [29–32]

## PKC isozymes expression in the heart and various tissues

PKC isozymes are ubiquitously expressed in all tissues at all times of development. Extensive experimental research indicates that different PKC isoforms serve distinct biological functions [27, 33–35]. Interestingly, it has been observed that PKC isoforms differ in their tissue distribution. Analysis, using Northern blotting immune-blotting techniques, revealed that many isozymes are widely expressed in a variety of tissues, while others are only expressed in a few tissues. In addition to their tissue distribution, PKC isoforms have been shown to differ with respect to substrate specificity [27, 36–40] and their susceptibility to downregulate upon phorbol ester treatment [9]. Several studies have revealed that there exist distinct individual functions in vitro studies among PKC isoforms. Examples of such isoforms include PKC-α and PKC-β phosphorylate histone IIIS strongly, while the other isozymes do so weakly, if at all [27]. Johnson et al. [34] investigated the spontaneous rate of contraction of neonatal rats and found that myocytes can be inhibited by activation of PKC-ε, but not by PKC-α, PKC-β, PKC-δ, or PKC-ζ. Studies relating to in situ binding of individual PKCs to specific intercellular proteins have not been well investigated.

Knowledge of the expression of PKCs in tissues is an important factor to help in understanding which PKC isozymes are involved in specific cardiovascular functions. PKCs have demonstrated to have sometimes opposing roles in both normal and diseased states [41], and Basu et al. [42] have shown that depending on stimulation, they can have opposing roles in the same cell. The relative content of each isozyme in the heart has been a controversial issue since it was found as different in different species. Numerous studies have investigated PKC expression pattern in cardiac tissues from various mammalian species including rats [43–52], rabbits [53–55], guinea pig [56], hamster [57], and dog [58].

Initial research by Hug et al. [59] showed that PKC-α, PKC-β, PKC-δ, PKC-ε, PKC-λ, and PKC-ζ were found to be widely distributed in many tissues, including the muscle, brain, lung, skin, and heart. Studies indicate [14] that PKC-θ is mainly expressed in the skeletal muscle, platelets, haematopoietic cells, and endothelium. In one of the first reports characterizing the expression of PKC isoenzymes in the heart, PKC-ε was described as the principal, if not the only PKC isoenzyme to be expressed in the rat heart [51]. Khalil et al. [60] and Liou et al. [61] reported the presence of PKC-(α, β, δ, ε, ζ) in vascular smooth muscle, while these isoenzymes, in addition to PKC-η and PKC-θ, were found to be expressed in endothelium platelets. Later, many studies identified the presence of PKC-α, PKC-δ, PKC-ε, PKC-η, and PKCs-ζ in rat-cultured cardiomyocytes [62–64], and even PKC-γ that was considered to be present only in the nervous system and adrenal tissues was found in the rabbit heart [32, 54]. Abundant expression of both βI and βIIPKC in human cardiomyocytes has also been reported [38, 65–68]. However, with the vast amount of studies on animal hearts, there exist only few reports available that examined the expression of PKCs in human hearts.

Bowling et al. [66] identified expression of PKC-α, βI, βII, and ε in human heart tissues using antibodies by Western blot analysis. Work done by Shin et al. [68] represented the first comprehensive study of PKC isoform expression in human ventricle, utilizing antibodies directed against all known PKC isoforms. The findings from their work, performed by Western analysis and immune-histochemistry, revealed that all isoforms, except PKC-ϒ and PKC-θ, were detected, indicating that in human ventricular myocytes, PKC expression is remarkably diverse. The findings of another study carried out by Simonis et al. [67], using polyclonal antibodies/monoclonal antibodies by Western blot analyses technique, revealed that in the human heart, six isoforms of PKC are expressed. These are PKC-α, PKC-β, PKC-δ, PKC-ε, PKC-λ, and PKC-ζ. PKC-γ and PKC-θ were not present in the human heart, consistent with previous finding. This study also highlighted the importance in relative distribution between atria and ventricles. PKC-ζ and PKC-δ are primarily expressed in the atria, while PKC-α, PKC-βI, and PKC-βII, which are all Ca^2+^-dependent, reside predominantly in the ventricle. PKC-ε and PKC-λ are evenly distributed in both atria and ventricles.

## PKC isozymes in cardiac diseases and heart failure

In addition to roles in regulations, alterations in PKC levels are associated with normal cardiac development. PKC-α, PKC-β, PKC-ε, and PKC-ζ expressions are high in foetal and neonatal hearts but decrease in expressions in adult hearts [69]. However, it was shown [66] that during the process of heart failure in humans, the levels of PKC-α, and PKC-β isozymes increase.

Mounting evidence suggests, and it has also been observed that individual or multiple PKCs are involved in cardiac diseases and heart failure [69]. These include, but not limited to, atherosclerosis [70], myocardial infarction, acute ischaemic [30, 55, 71–73], cardiac hypertrophy [29, 74], cardiac arrhythmia [75], heart failure [76], and cardiac fibrosis [77].

### Myocardial infarction and ischaemia preconditioning

Ischaemic heart disease continues to be the leading cause of death in Western countries. Over the past two decades, significant effort, especially with preliminary work done by Ytrehus et al. [78], has been devoted in understanding the role of specific PKCs in cardiac diseases. Preconditioning can be described as a natural cardiac-protective mechanism, and it involves subjecting the heart to brief periods of ischaemia and reperfusion prior to a longer ischemic period. Preconditioning protects the heart from ischaemia and reperfusion-induced damage [79] by inducing myocardial adaptation to the ensuing prolonged ischemic event. In other words, a brief period of ischaemia followed by reperfusion renders the heart more resistant to injury from a subsequent longer ischaemic insult instead of accentuating the injury. These results, using a canine heart, were some of the first to highlight the fact that direct PKC activation prior to ischaemic event provides cardiac protection. Based upon seminal observations from these experiments, the term ischemic preconditioning (IPC) was used to describe this phenomenon. IPC was subsequently shown to be effective in other species including rats [80], sheep [81], rabbits [82], and pigs [83].

The role of PKCs in ischemic preconditioning is now well established in a variety of mammalian models including rats [84–86], rabbits [78, 87], and canine [88] where different specific PKCs have been found in various animal species. However, although the complex choreography of activation or inhibition of various isoforms of PKC with ischaemia and reperfusion has been worked out in animal models over the past 30 years, there has been no success in translating this knowledge into useful therapy in humans.

Research work by Yellon et al. [89] was one of the first studies of IPC in humans where they examined whether a preconditioning protocol protects the myocardium from prolonged ischaemia. Their study showed that preconditioning ultimately leads to a preservation of ATP levels in preconditioned human hearts in contrast to non-preconditioned hearts. Subsequent studies by Yellon et al. [90–93] and others [94–96] provided further evidence for PKC involvement in human IPC.

The mechanism of preconditioning is still a subject of debate. One of the earlier favoured hypotheses for preconditioning suggests that endogenous ligands such as adenosine initiate an intracellular pathway by acting on G protein-linked receptors leading to the activation of PKC via diacylglycerol [78]. After which, activated PKC then phosphorylates a secondary effector protein, which is thought to induce protection.

There have been supportive [97] and conflicting reports [98, 99] with respect to the role of PKCs in ischaemic preconditioning. It was even suggested [100] that PKC might rather be a ‘spectator’ rather than a ‘player’, that is, it seems likely that PKC activation is an epiphenomenon rather than a mandatory or exclusive means of preconditioning the heart. Subsequent studies suggest that cardiac preconditioning inhibits both apoptosis and necrosis [101]. Earlier conflicting data were related to the initial use of non-selective individual PKC activators/inhibitors such as diacylglycerol (DAG), indolocarbazole, and bisindolymaleimides [102, 103] that exhibited poor selectivity for PKC isozyme. Subsequent studies, using selective isozyme-specific inhibitors and activators (6–10 amino acids in length), helped to explain earlier reported uncertainty.

### Translating ischemic conditioning from animal models to human

While on the topic of IPC, it is important to briefly discuss challenges of translation of cardioprotection, its limitations in human studies, and need for PKC manipulation in ischaemia/reperfusion. Translation of cardioprotection can be characterized as a four-step process from (1) reductionist animal studies to (2) more clinically relevant animal studies, to (3) clinical proof-of-concept studies with surrogate end points such as infarct size, and to finally (4) clinical outcome trials [104].

Since the first clinical study conducted to test external application of an IPC stimulus in patients undergoing coronary artery bypass graft (CABG) surgery, more than 150 clinical trials have been conducted and thousands of experimental animal studies on mechanical and pharmacological conditioning and cardioprotective interventions. However, the concept on the translation of cardioprotection strategies to clinical practice continues to disappoint. There is yet no single randomized clinical trial, which has explicitly demonstrated a better clinical outcome for patients experiencing an acute myocardial infarction or undergoing cardiovascular surgery when receiving an adjunct cardioprotective.

In the field of cardioprotection, substantial gaps still remain between experimental studies aiming at the identification of novel mechanisms and studies providing robust preclinical data that are worth of being tested in humans.

The critical time frame for adjunct cardioprotection that depends on factors such as (1) species (2) heart rate, and (3) residual blood flow still constitutes a major problem [105]. Systematic animal studies on the time frame for adjunct cardioprotection, in interaction with the above listed variables, are lacking while the exact time frame for adjunct cardioprotection in humans is not really clear.

A very important fact when extrapolating from animal models to humans is that it is vital to understand the differences between animal models and patients. Most animal experiments, including larger mammals that are closer to humans in their anatomy and physiology, are performed in young and healthy animals that lack the risk factors. Compare this to older individuals with cardiovascular disease who participate in clinical trials, with comorbidities such as diabetes, hypertension, kidney disease, and are taking medications [106]. Secondly, the effectiveness of ischemic-conditioning strategies in humans seems to be less profound than reported in the animal literature, with some randomized clinical trials showing no significant benefit [107, 108]. These disparities are keys to understanding why ischemic-conditioning strategies fail to translate from animals to humans.

The results of large, multi-centre, randomized, controlled clinical trials of ischemic conditioning on clinical outcomes after cardiac surgery have highlighted the challenges in translating cardioprotection into clinical practice. In future, it is recommended that only results that have been proven robust in multi-centre approaches be worth tested for translation to patients.

With respect to PKC kinases and translation cardioprotection, investigation of signalling pathways underlying ischemic conditioning has identified molecular targets for pharmacological manipulation—a therapeutic strategy termed ‘pharmacological cardioprotection’. The PKC family of kinases plays essential roles in CVDs and has been linked as playing an important role in the reperfusion injury salvage kinase (RISK) pathway in IPC mechanism. Since this technique of pharmacological manipulation was realized, there has been much excitement on the role of kinases in PKC manipulations in IPC. However, over time, it has been revealed that there does not appear to be any translational-clinical science benefit on the horizon for manipulation of PKC in ischaemia/reperfusion. This currently disappointing situation has led many clinicians to prematurely give up on attempts of PKC pharmacological cardioprotection beyond rapid reperfusion with more focus being placed on long-term cardiovascular therapies.

### PKC-δ and PKC-ε in myocardial infarction, ischemic reperfusion, and preconditioning

Although they are members of the same subgroup (the so-called novel group), PKC-δ (commonly referred to as pro-death kinase) and PKC-ε (commonly referred to as pro-survival kinase) mediate contrasting and even opposing effects. They are both activated in the ischaemic human heart [109] where they play a key role in ischaemic preconditioning. However, the mechanism and exact role of PKC in the survival of cardiac cells remain unknown and controversial with research confirmed that these two related PKC isozymes have both parallel and opposing effects in the heart, indicating the danger in the use of therapeutics with non-selective isozyme inhibitors and activators [110]. Studies by Hassouna et al. [65], using various specific PKC inhibitors, investigated which PKCs were involved in IPC of the human atrial myocardium sections using the temporal relationship to the opening of mitoK_ATP_ channels. The results, with reference to PKC-δ and PKC-ε, showed that PKC-ε inhibitors blocked IPC of the human myocardium and is upstream of mitoK_ATP_ channels while PKC-δ inhibitors did not blocked IPC.

**Fig. 2:**
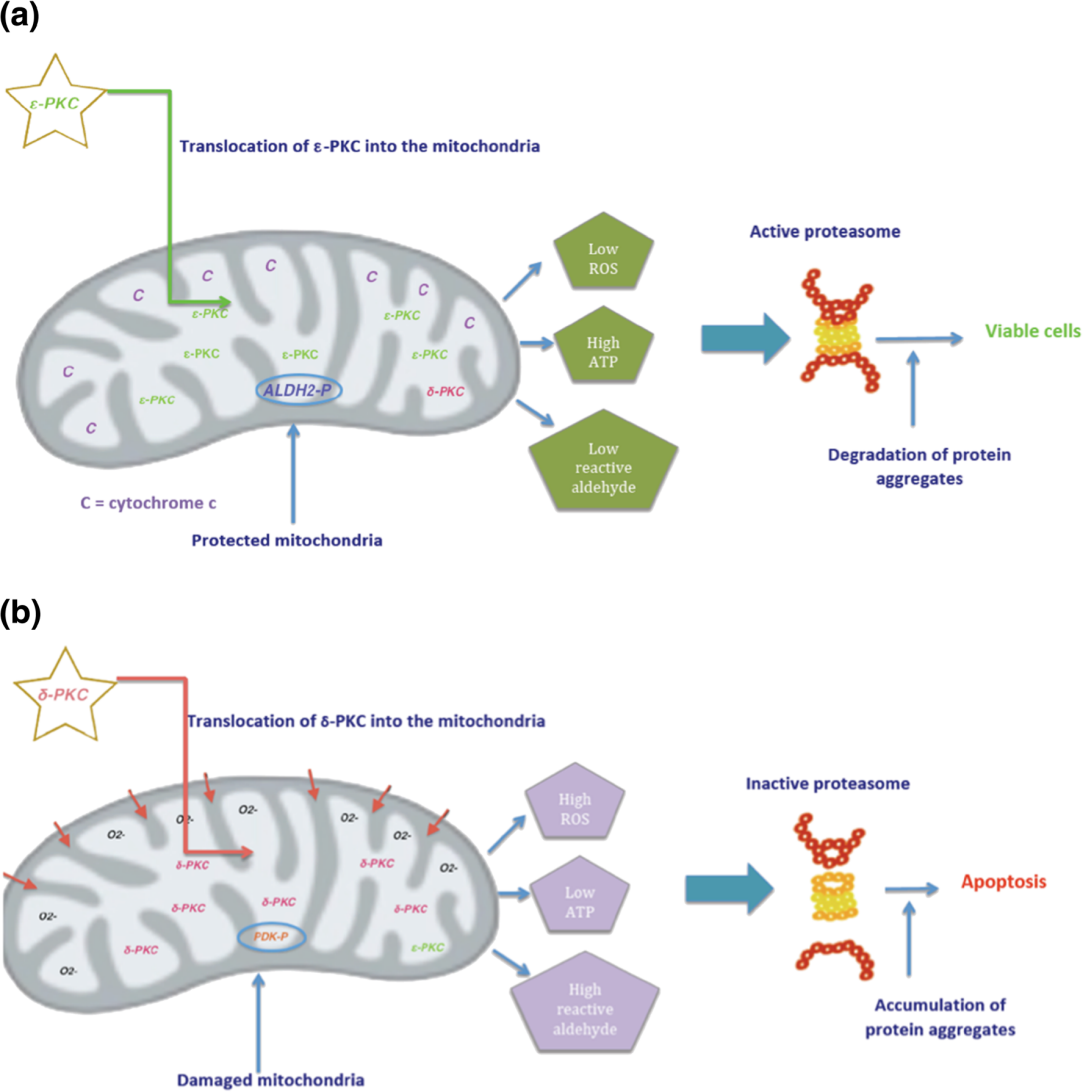
The role of PKC isozymes in ischaemic heart disease. Schematic diagram showing **a** how ischaemic preconditioning prior to prolonged ischaemia and reperfusion provides cardioprotection by activating more PKC-ε, which translocate into the mitochondria and prevents mitochondrial dysfunction induced by prolonged ischaemia and reperfusion. **b** In contrast, prolonged ischaemia and reperfusion result in activation of PKC-δ more than PKC-ε, leading also to translocation of PKC-δ into the mitochondria. Mitochondrial dysfunction and increase in ROS lead to both apoptosis and necrosis and severe cardiac dysfunction

Ischaemia and reperfusion cardiac damages have shown [111, 112] to be dependent on translocation of PKC-δ into the mitochondria where cytochrome c is released resulting in inhibition of mitochondrial functions. It has been suggested that oxidative stress seems to trigger PKC-δ into the mitochondria [113]. PKC-δ activation results in phosphorylation steps [114] and inhibition of ATP regeneration. Cardiac mitochondrial inhibition now triggers higher reactive oxygen species (ROS) production and built up of reactive aldehydes (e.g. 4-hydroxynonenal (4-HNE), methylglyoxal (MGO), and others), which can become toxic at accumulated levels [115]. With a combination of diminished levels of ATP, accumulated of ROS, and toxic aldehydes, this results in accumulation of aggregated proteins and an inactive 26S proteasome, ultimately, leading to both apoptosis and necrosis [41] followed by severe cardiac dysfunction. It is no surprise, as numerous research studies have now shown, that PKC-δ inhibition will result in opposite effects to that of its activation. That is, PKC-δ inhibition at reperfusion is protective (refer to schematic diagrams in Fig. 2a, b).

Additionally, it is now recognized that IPC consists of two (2) chronologically and patho-physiologically distinct phases comprised of an early phase and a late phase of protection. Stein et al. [116] have reported that PKC-ε activation facilitates the protective effects of late preconditioning. That is, precondition stimuli enhance the resistance of the heart to ischaemia injury 12–72 h later.

Inagaki et al. [117] have shown that PKC-δ inhibition reduces reperfusion injury to the myocardium by inhibiting both apoptosis and necrosis. Further work [110] using selective peptide inhibitors (δV_1–1_) has demonstrated that inhibition of PKC-δ protects the heart from ischaemic injury and further, PKC-δ activation is cardioprotective provided that there is sufficient time allowed for PKC-ε activation. These findings are in accordance with a role for PKC-δ in apoptosis as previously demonstrated by overexpression of PKC-δ [118]. It has been suggested also that inhibition of PKC-δ should be a target for drug development to prevent irreversible cardiac injury during reperfusion in humans.

Interestingly, it has been reported [109] that activated PKC-δ has two potential fates that, apparently, depend on the metabolic rate of the cell. These include (1) if the integrity of the 26S proteasome, mitochondrial function, and cellular energy is maintained, PKC-δ is effectually degraded and (2) conversely, if the aforementioned parameters are not maintained, the result is an accumulation of elevated levels of activated PKC-δ (pro-death kinase) in the mitochondria.

PKC-ε isozyme is also translocated into the mitochondria by stimuli; however, its activation has shown (in contrast to PKC-δ) to be protective [110, 119, 120] when occurring just before reperfusion. In addition, it also prevents mitochondrial dysfunction induced by prolonged ischaemia events. Mitochondrial protection is achieved by PKC-ε phosphorylation followed by activation of aldehyde dehydrogenase 2 [ALDH2]—which removes the harmful aldehyde (4HNE; MGO) and peroxidation by-products [115]. Research work by Chen et al. [121] showed that this mitochondrial enzyme (ALDH2) correlated with reduced ischaemic heart damage in rodent models, in some cases, reduced infract size by 60%. The above steps now result in lower ROS concentration, promote faster recovery of ATP and faster removal of aggregated proteins, promote an active 26S proteasome, and ultimately result in reducing cellular damage—diminished apoptosis and necrosis. In addition to the aforementioned factors and cardio benefits from PKC-ε activation, upon translocation into the mitochondria, PKC-ε isozyme is involved/initiated a number of processes that help to contribute in overall cardioprotection. These include, but not limited to, the following: firstly, opening of K_ATP_ channels—this channel opens as ATP levels fall and is inhibited when levels are high. The K_ATP_ channel, which exists in both mitochondria and sarcolemmal membrane has been recognized by Gross et al. [122] to be a likely end effector of ischaemic preconditioning, and earlier work [123] suggests that the sarcolemmal channel surface might be an important effector of the cardioprotective effects of ischemic/hypoxic preconditioning. Secondly, restrict mitochondria permeability transition pore (MPTP) from opening—this pore has been identified by Bains et al. [124] and others [125] as an effector of preconditioning. PKC-ε interacts with and inhibits the MPTP and, thus, stabilizes mitochondria in cardiac tissue during and following ischaemia. This pore, which allows water and solutes to enter the mitochondria, is closed during ischaemia and opens in the first few minutes of reperfusion [126]. It ultimately inhibits the pathological function of the pore and contributes to PKC-ε induced cardioprotection. Thirdly, increase in cytochrome c activity—PKC-ε co-immuno-precipitates with cytochrome oxidase subunit IV and is associated with improved cytochrome c oxidase activity and cardioprotection [127]. Finally, it was reported [109] that the active proteasome that results from PKC-ε activation is capable of selectively degrading activated PKC-δ, thereby altering the ratio between PKC-ε and PKC-δ, increasing the favour of pro-survival kinase (PKC-ε), and ultimately regulating myocardial sustainability.

### Cardiac hypertrophy and heart failure

Cardiac hypertrophy is a thickening of the interventricular wall and/or septum in the cells and it involves complex multiple progressive alterations of the heart geometry in response to either mechanical, electrical, or neuro-humoral stimuli such as epinephrine, norepinephrine, aldosterone, and angiotensin II. It may be further characterized with increase in cardiomyocyte size, increased protein synthesis, and changes in the organization of the sarcomeric structure. Although short-term subcellular changes (cardiomyocyte enlargement, formation of new sarcomeres, etc.) associated with cardio hypertrophy may be beneficial, however, when sustained for longer intervals, the cardiac system becomes maladaptive. This eventually leads to decompensation resulting in fibrosis, apoptosis, and cardiac remodelling among other cardiac diseases before transitioning to heart failure. Hypertrophy is therefore an early indication during clinical course of heart failure and plays an important risk factor for subsequent cardiac death.

Cardiac hypertrophy can be placed into three categories—(1) normal growth, (2) growth induced by physical condition, and (3) growth induced buy pathological stimuli—and various kinases have been identified as mediators in response to activation induced by neuro-hormone receptors [128].

Protein kinase C family have been identified as having important roles in adaptive and maladaptive cardiac responses. In cultured myocytes, it has been found that PKCs regulate contractibility and hypertrophy [128]. Studies have identified the intercellular mechanism underlying cardiac hypertrophy and PKC isozymes as potential mediators of hypertrophic stimuli [76, 129], and it has also been suggested that induced stress associated with cardiac hypertrophy coupled with PKC activation will increase PKC expression and activity [130]. As previously mentioned, PKC expression in cardiac tissue differs with species, cell type, and developmental stage. Importantly, the activity of PKCs is dependent upon its localization within the cell, expression level, and phosphorylation [131]. The following chapter focuses on the role of PKC isoforms in the aetiology of cardiac hypertrophy and heart failure.

### PKC-δ and PKC-ε in cardiac hypertrophy and heart failure

In contrast with preconditioning to ischaemia in which PKC-δ and PKC-ε have opposite roles, both act in the same direction during the development of hypertrophy [110]. The activation of PKC-ε may be a factor that induces ventricular hypertrophy with its positive effect on cell growth. In this line, the relation between PKC-ε and the cytoskeleton is a mechanism that potentially initiates hypertrophy via phosphorylation of proteins in the costameres, which then transmit signalling to the Z-disk for parallel or series addition of thin filaments regulated via actin capping [132].

The activation of PKC-ε was shown during stretch of cardiomyocytes [133]. In isolated guinea pig hearts, stretch, one of the principal activators of ventricular hypertrophy, has been shown to induce a PKC-ε translocation to membranes that was partially inhibited by losartan [56]. In vivo, an induction of concentric cardiac hypertrophy with an overexpression of constitutively active PKC-ε [134] or with the expression of cardiac specific PKC-ε activator [135] was shown. The effect of PKC-ε is in general considered to lead to a concentric hypertrophy. However, in mice overexpressing PKC-ε [136], the evolution of hypertrophy was quite deleterious since it led to a dilated cardiomyopathy. Thus, the effect of PKC-ε may differ depending upon its level of expression.

PKC activity has been generally described as increased with different behaviours of different isozymes. In general, PKC-ε and PKC-δ increased content and translocation towards the membrane fraction was found but this is not a universal finding in all types of hypertrophy. In aortic banding in rats [137], guinea pigs [138], and in severe human aortic stenosis [67], an increased concentration of PKC-ε was found in membranes. In contrast, PKC-δ content was found as unchanged in nuclear-cytoskeletal fraction in the model of rat aortic banding [137]. Other researchers found the same translocation in a completely different type of hypertrophy, right ventricular hypertrophy induced by pulmonary hypertension due to chronic hypoxia in rats [139]. However, opposite results were described in hypertrophy or heart failure by others [66, 32, 140–142]. In human failing hearts, left ventricular PKC-ε content was decreased [67]. In rabbit left ventricular hypertrophy, researchers have found a decreased cardiac content of PKC-ε and a similar downregulation was demonstrated in a model of genetic hypertension while PKC-δ was unaffected [141]. In contrast, PKC-ε activity was found to be unchanged in rat aorto-caval fistulas while PKC-δ was increased [142]. Although PKC-ε is an actor in the development of hypertrophy, its expression in the myocardium and its translocation are not found as increased in all models. More recently, it has been suggested that PKC-ε inhibition attenuates pathological remodelling in hypertension-induced heart failure by preventing cardiac mast cell degranulation [143].

PKC-ε has been shown to bind scaffold proteins. In the heart, F-actin bound PKC-ε selectively over PKC-δ [144] and it was shown that the binding interface between PKC-ε and cardiac myofilaments was mainly on the V1 region of PKC-ε and the interface between PKC-ε and F-actin was mainly on the C1 region of PKC-ε [144].

### PKC-β in cardiac hypertrophy and heart failure

PKC-β was chosen as the first isoenzyme [145] to be studied using cardiac target expression and has been shown to play an important role in cardiac hypertrophy. One of the main reasons for this being is its reactivity and expression increases in human heart failure [67]. The result showed that the calcium dependant PKC-β (stained as a triple band containing both splice variants {PKC-β1 and PKC-β11}) resides predominantly in the ventricular myocardium. They also demonstrated that in downregulation during ontogenesis in human hearts, PKC-β expression was decreased by 90%—that is, this isoform is almost totally switched off in normal adult non-failing cardiac heart. PKC-β is highly upregulated, leading to re-expression in dilated cardiomyopathy originating from severe heart failure.

Using explanted heart from patients in whom dilated cardiomyopathy was diagnosed, Bowling et al. [66] examined PKC isoforms present in these samples. Their results showed a quantitative increase of >40% in PKC-β1 and PKC-β11 membrane expression in failed human hearts compared with non-failed hearts. They also reported a reduction in membrane activity from failed hearts of 209 pmol min^−1^ mg^−1^ when a selective PKC-β inhibitor (LY333531-macrocyclic bis maleimide) was used (compared with 45.2 pmol min^−1^ mg^−1^). An important conclusion in the finding from their research is that in failed human heart, PKC-β1 and PKC-β11 expression and contribution to the total PKC activity are significantly increased.

### PKC-α in cardiac hypertrophy

With respect to the conventional isoforms, PKC-α is the predominant subtype expressed in the mouse, human, and rabbit hearts, while PKC-β and PKC-γ are detectable but expressed at substantially lower levels [138, 146, 147]. Although it is the most highly expressed of the myocardial PKC isoforms, PKC-α is the least studied because unlike PKC-δ and PKC-ε, it is not regulated in acute myocardial ischaemia [148] and in contrast to PKC-β, it is not regulated in diabetes [145]. Reports have associated PKC-α activation or an increase in PKC-α expression with hypertrophy, dilated cardiomyopathy, ischaemic injury, or mitogen stimulation [128]. Increased expression of PKC-α was also observed following myocardial infarction [67]. Human heart failure has also been associated with increased activation of conventional PKC isoforms, including PKC-α [66, 67]. Thus, PKC-α fits an important criterion as a therapeutic target; its expression and activity are increased during heart disease. Initial comparative analysis of PKC isoforms [149] using wild-type or dominant inhibitory forms of PKC-α, PKC-β2, PKC-δ, and PKC-ε suggested that only PKC-α was sufficient to stimulate cell hypertrophy and only inhibition of PKC-α inhibited agonist-mediated hypertrophy. The implication of this work [149] was that PKC-α is a key regulator of cardiomyocyte hypertrophic growth.

The concept that PKC-α is of a greater importance as a regulator of myocardial contractility vs. cardiac hypertrophy was highlighted by Hahn et al. [150] using RACK binding and pseudo-RACK peptides derived from PKC-β. Previous studies [151, 152] have demonstrated that chronic activation of PKC-α diminished baseline ventricular ejection performance and, in combination with Gq-mediated hypertrophy, caused a lethal cardiomyopathy. In contrast to this, chronic PKC-α inhibition improved myocardial contractility and inhibited Gq-mediated cardiac hypertrophy [150]. The results of these studies showed that PKC-α is a critical determinant of myocardial systolic function but has minimal effects on cardiac hypertrophy.

### Cardiac fibrosis

Cardiac fibrosis is the accumulation of fibroblasts that result from the expansion of the cardiac extracellular matrix proteins such as collagen, by augmented release from fibroblasts or reduced degradation of collagen. Cardiac fibrosis is crucial for scar formation after acute myocardial infarction (AMI). Ischemic injury results in increased levels of circulating cytokines, growth factors, and hormones that stimulate cell surface receptors on cardiac fibroblasts.

Fibrosis reduces the flexibility of myocardial tissue resulting in diastolic dysfunction, leading to myocardial malfunctioning (increased thickening of extracellular matrix, decreased cardiac elasticity), and consequently posing detrimental effects to failing hearts. Additionally, increased collagen content disrupts electrical connectivity between cardiomyocytes resulting in arrhythmogenesis [153].

### Role of PKC isozymes in cardiac fibroblast proliferation

PKC isozymes contribute to different stages of cardiac fibroblast proliferation [153–155]. Fibroblast adhesion to the extracellular matrix has shown to be regulated through PKC-ε (via βI-integrin) while upregulation of cytokine and growth factors are mediated by PKC-α, PKC-βII, PKC-δ, PKC-ε, and PKC-ζ. In addition, PKC-δ, PKC-ε, and PKC-ζ have been demonstrated to regulate fibroblast proliferation with PKC-δ and PKC-ζ yielding opposing results in fibroblast [156].

PKCs also regulate activity and concentrations of matrix metalloproteinase (MMP), which facilitate the motility of cardiac fibroblast [157, 158]. It has been demonstrated that PKC-θ and PKC-ζ increase activities of both MMP-2 and MMP-9 via ERK pathways in cardiac fibroblast [151]. However, in the JNK-dependent pathway, PKC-α and PKC-βI increase activity of MMP-9 and not MMP-2 [159].

Additional research that focused specifically on the critical role of PKC-ε in mediating cardiac fibrosis and the results has yielded promising insight. Mechanistic studies have demonstrated that PKC-ε forms a tight complex with β1-integrin to regulate the interaction between the cell and extra cellular matrix ECM [160, 161]. These findings help to validate a role of PKC-ε in mediating cardiac fibroblast adhesion.

### Atherosclerosis

The hallmark of coronary heart disease is characterized by the development of endothelial dysfunction followed by atherosclerotic (thickening of artery wall as a result of invasion and accumulation of white blood cells) lesions in the coronary arteries leading to sustained ischaemic events and acute myocardial infarction (AMI). Atherosclerosis progression begins with low-density lipoprotein (LDL) accumulation followed by monocyte- and endothelium-mediated oxidation of LDL, monocyte extravasation, foam cell formation, and finally, formation of atherosclerotic plaque.

The role of PKCs has been shown to be intimately involved with various stages of atherosclerotic progression. Studies on human hepatic G2 cells, U-931 (human histiocytic lymphoma), and human endothelium have demonstrated isozyme-specific effects of PKC with different stages in atherosclerotic progression [162–171]. The effects and roles of PKCs in atherosclerosis and heart failure in human heart are summarized in Table 1.

**Table 1:** Table showing the role of isozyme-specific PKCs in human heart failure and atherosclerosis

PKC	Cardiac aetiology	Model	Features	Ref.
PKC-βII	Heart failure	Human end-stage dilated cardiac myopathy	Increase cardiac PKC-βII levels	[67]
PKC-βII	Heart failure	Human end-stage dilated cardiac myopathy	Increase cardiac PKC-βI levels	[66]
PKC-α	Atherosclerosis	Human endothelium	Increases superoxide production and inactivation of PKC-α	[162]
PKC-α	Atherosclerosis	HepG2	LDL oxidation and decreased superoxide	[163]
PKC-α	Atherosclerosis	U-937 cells	PECAM1 expression and adhesion	[164]
PKC-α	Atherosclerosis	Human endothelium	Increased MMP-2 expression	[165]
PKC-α	Atherosclerosis	HepG2	LDL upregulation	[166]
PKC-β	Atherosclerosis	HepG2	Increased LDL activity	[167]
PKC-β	Atherosclerosis	Human endothelium	Induces expression of vascular cell adhesion, translocation of PKC-β	[159]
PKC-β	Atherosclerosis	Human endothelium	Increased MMP-1 and MMP-3 expression	[161]
PKC-β	Atherosclerosis	Human endothelium	Increased MMP-2 expression	[165]
PKC-ε	Atherosclerosis	HepG2	Increased/decreased LDL activity	[170]
PKC-ε	Atherosclerosis	Human endothelium	Induces expression of vascular cell adhesion, translocation of PKC-β	[165]
PKC-ζ	Atherosclerosis	Human endothelium	Regulates TNF-α-induced activation of NADPH oxidase	[171]

## PKC—a target for drug development

The PKC family of kinases plays essential roles not only in CVDs but also in other diseases. This makes them an attractive target for drug development. This section will discuss areas for future investigation that may lead to drug development and novel therapeutic approaches.

The idea of PKCs as target for drug development dates back to the early 1980s, when they were first identified as the receptors for the tumour-promoter phorbol ester [172]. The central role of PKCs as tempting target for drug development is associated with the fact that these kinases are activated in a variety of diseases as evidenced in animal models and human tissue studies. In addition to heart failure and heart diseases that were extensively covered in previous sections of this review, evidence exist for the critical role of PKC in cancer [173], diabetes [174], bipolar disease [175], Parkinson’s disease [176], Alzheimer’s disease [177], psoriasis [178], kidney [179], and many other human diseases.

Researchers have been trying for years to develop PKC-specific inhibitors that are isozyme selective. Various approaches have led to development of ATP-competitive small molecules (targets the catalytic domain) [180], activators and inhibitors that mimic DG-binding (targets C1 domain) [181], and protein–protein interactions between regulatory region and RACK [182].

PKC modulation in human diseases has shown great promise but sadly, clinical trials’ results have been disappointing. Trials include transplantation clinical trial [183], bipolar disorder trials [184], oncology trials [185], diabetic trials [186], and cardiovascular trials [187]. The major challenges in clinical application of PKC modulators are due mainly to unforeseen adverse reactions, inadequate therapeutic effect, insufficient preclinical studies, absence of blood biomarkers, and lack of selectivity (PKC inhibitors also affect other kinases). Table 2 provides a summary of clinical trials with PKC regulators in human diseases.

**Table 2:** Table showing summary of clinical trials of PKC regulators in various human diseases

Disease	Drug	Mechanism	Ref.
Transplant rejection	Sotrastaurin	↓PKC	[183]
Bipolar mania	Tamoxifen	↓PKC (at high dose)	[184]
Diabetic retinopathy	Ruboxistaurin	↓PKC-β	[186]
Oncology	Aprinocarsen Bryostatin Enzastaurin Midostaurin Tamoxifen	↓PKC-α ↑PKC ↓PKC-β ↓PKC ↓PKC (at high dose)	[188] [185, 189] [190, 191] [192] [193, 194]
Congestive heart failure	Flosequinan	↓PKC	[187]
Coronary bypass grafting	Volatile anaesthetics Adenosine Acadesine	↑PKC-ε ↑PKC-ε ↑PKC-ε	[195, 196] [197, 198] [199, 200]
Acute myocardial infarction Salvage	Adenosine Delcasertib	↑PKC-ε ↓PKC-δ	[201] [202]

With all the excitement around PKC as targets for drug development, both academic and pharmaceutical efforts have failed to produce a single new drug that specifically targets PKC.

A future direction for drug development has been linked to post-translational modification of PKC, based upon secondary messenger-dependent activation. These modifications include tyrosine nitration, tyrosine phosphorylation, N-acetylglucosamine O-linked (O-GlcNAc) to serines and threonines of cytosolic and nuclear proteins, oxidation of cysteine rich domain within the C1 domain, and proteolytic cleavage of the enzyme at the hinge region between the catalytic and the regulator halves [203].

Post-translational modification represents a ubiquitous and essential device for control of protein activity, localization, stability, and protein–protein interaction. The importance of this is further emphasize by the fact that covalent post-translational modification, namely serine/threonine phosphorylation of PKC along with binding of PKC to the lipid second messenger diacylglycerol, is recognized as two equally important mechanisms that regulate

PKC activity [204]. About 100 mammalian proteins, including signalling components, metabolic enzymes, and transcription factors, have been identified that carries this modification [205, 206]. However, while the modification has been known for over 30 years, and provides an alternative means of PKC activation which may play a role in disease states, no pharmacological agents have been developed yet based on second messenger-independent activation of PKC.

The PKC family still remains a desirable target for drug development. Biomarkers for specific PKC activity will play a major role for future success in developing drugs for PKC-mediated disease. There is also the need for greater and efficient drug development practices and adequate preclinical studies.

## Conclusion

In conclusion, this review provides a comprehensive description of the structure, functions, and distribution of PKCs in the healthy and diseased heart with some emphasis on human heart. The study further focuses mainly on their regulation and roles in the normal healthy heart and, more so, their involvement in the development of heart failure. The regulation of the different isozymes of PKC by pharmaceutical agents may have potential benefits in the treatment of heart failure, thereby promoting a better quality of life for the patients.
